# Modifiable Risk Factors Affecting Outcomes: The Truth About Obesity

**Published:** 2014-09-01

**Authors:** Pamela Hallquist Viale

The American Society of Clinical Oncology (ASCO) held its annual meeting this past June. As usual, participants had the opportunity to learn and discuss the latest in oncology research findings. Although I didn’t attend the meeting in person, I noted the daily reports with interest. I was particularly appalled by the results of a study examining 80,000 patients in early breast cancer trials and their outcomes based on several factors, including obesity.

## Obesity and Its Effect on Early Breast Cancer

Pan & Gray, (2014) reported on the effect of obesity in premenopausal estrogen receptor (ER)-positive early breast cancer using Early Breast Cancer Trialists’ Collaborative Group data from 70 trials. The authors utilized the data to determine body mass index (BMI), ER and menopausal status, and cancer recurrence and prognosis. They noted that obesity (defined as BMI > 30 kg/m²) has been associated with a poorer prognosis in this patient population (Pan & Gray, 2014).

The results demonstrated that in 20,000 women with ER-negative disease, there was a minimal association between BMI and breast cancer mortality, but that in 60,000 women with ER-positive tumors, BMI was associated with disease mortality in pre- and perimenopausal women, as well as postmenopausal women (each 2*P* < .00001). After the investigators adjusted for additional tumor aspects, the positive association was clearly significant in only 20,000 of the pre- and perimenopausal women with ER-positive breast cancer, demonstrating an increased risk for worse outcomes, including death. When subdivided by age, obesity was significant only to approximately age 55.

Most clinicians are strongly aware of the risks of smoking and the many different types of cancer associated with that habit, yet most of us do not completely understand obesity and cancer risk. However, a 10-year-old study published in the *Journal of the National Cancer Institute* highlighted the breast cancer risk for women based on increased BMI, stating that this risk was largely the result of increased serum concentrations of bioavailable estradiol stemming from obesity (Key, 2003).

## Obesity as a Worldwide Problem

It is especially disconcerting to see that the prevalence of obesity in several countries is increasing. The phenomenon has been termed a global epidemic, causing an estimated 3 to 4 million deaths in 2010 (Ng et al., 2014). Obesity is linked to a myriad of health problems, including cardiovascular disease, diabetes, osteoarthritis, and chronic kidney disease, as well as cancer (Ng et al., 2014). Recently reported research on worldwide obesity showed that the prevalence of overweight individuals has increased in at-risk populations of children and adolescents in developing countries; in some populations, the prevalence exceeds 50% (Ng et al., 2014).

## The Role of Advanced Practitioners

As advanced practitioners, we are charged with safely guiding our patients through newly diagnosed disease, implementing treatment protocols and managing symptoms throughout their illness and therapy. Survivorship is an equally important part of our care. Yet I can’t help but wonder whether we are doing a good job of educating our patients regarding the specific hazards of obesity. This recently reported research on premenopausal patients with ER-positive breast cancer should give us pause. Are we doing a thorough job of educating our patients regarding the true risk of obesity and its potential to affect overall outcomes from this disease? Can we treat our patients throughout their chemotherapy and hormonal therapies and neglect the importance of additional risk factors that can affect disease recurrence?

Advanced practitioners should arm themselves with the knowledge from this important study and engage in discussions about obesity with appropriate patients at the start of the diagnosis and treatment plan. Dietary assistance should be enlisted where appropriate, and milestones should be assessed at each visit. Obesity is a modifiable risk factor. As such, counseling regarding this factor should be considered an important part of the overall treatment protocol and survivorship plan.

## JADPRO Live at APSHO 2014

If you haven’t signed up to attend JADPRO Live at APSHO 2014, please do so! This conference will give you 3 days of focused education aimed specifically at the advanced practitioner’s level, plus an extra day of preconference workshops. The conference takes place October 30 through November 2, 2014, and will be held at the Loews Royal Pacific Hotel at Universal Orlando, Florida. You can view the conference agenda and preconference workshops at www.apsho.org/jadprolive. The conference offers the opportunity to earn up to 20 CE credits/contact hours for advanced practitioners in oncology, including nurse practitioners, physician assistants, pharmacists, physicians, and other providers. As this will also be the first official APSHO meeting, new members will receive a significant discount to the conference. I hope to see you there!
